# Apatinib combined with SOX regimen for conversion therapy in advanced gastric cancer patients: a retrospective cohort study

**DOI:** 10.1186/s12957-023-02973-3

**Published:** 2023-04-11

**Authors:** Ya-Ya Deng, Ding-Yi Jiang, Peng-Fei Zhu, Hongrui Lu, Qian Liu, Xinyue Zhang, Shuang-Yue Pan, Zhe-Ling Chen, Liu Yang

**Affiliations:** 1 Cancer Center, Department of Medical Oncology, Zhejiang Provincial People’s Hospital (Affiliated People’s Hospital, Hangzhou Medical College), Hangzhou, Zhejiang 310014 China; 2 The Qingdao University Medical College, Qingdao, Shandong Province 260075 China; 3 Graduate School of Clinical Medicine, Bengbu Medical College, Bengbu, 233000 Anhui Province China; 4 Graduate School of Clinical Medicine, Zhejiang Chinese Medical University, Hangzhou, 310014 Zhejiang Province China

**Keywords:** Apatinib, Gastric cancer, Conversion therapy, SOX

## Abstract

**Background:**

Recently, many studies have shown that the progress of conversion therapy can provide surgical opportunities for patients with advanced gastric cancer (GC) and bring survival benefits. However, the results of the current study show that the regimen used in conversion therapy is still controversial. Apatinib, as the standard third-line treatment for GC, has an inconclusive status in conversion therapy.

**Methods:**

This study retrospectively analyzed GC patients admitted to Zhejiang Provincial People’s Hospital from June 2016 to November 2019. All patients were pathologically diagnosed, had unresectable factors, and received SOX regimen with or without apatinib as conversion therapy.

**Results:**

A total of 50 patients were enrolled in the study. Altogether 33 patients (66%) received conversion surgery and 17 patients (34%) received conversion therapy without surgery. The median progression-free survival (PFS) between surgery group and non-surgery group were 21.0 versus 4.0 months (*p* < 0.0001), and the median overall survival (OS) were 29.0 versus 14.0 months (*p* < 0.0001). In conversion surgery group, 16 patients (16/33) were treated with SOX plus apatinib, and the R0 resection rate was 81.3%; 17 patients (17/33) were treated with SOX regimen along, and the R0 resection rate was 41.2% (*p* = 0.032). The PFS in the SOX combined with apatinib group was significantly longer than that of SOX group (25.5 versus 16 months, *p* = 0.045), and the median OS were 34.0 versus 23.0 months (*p* = 0.048). The addition of apatinib did not increase the incidence of serious adverse reactions throughout the preoperative therapy period.

**Conclusions:**

Patients with advanced inoperable gastric cancer could benefit probably from conversion chemotherapy and subsequence conversion surgery. Apatinib-targeted therapy combined with SOX chemotherapy may be a safe and feasible option for conversion therapy.

## Introduction

Gastric cancer (GC) is worldwide one of the most common cancers. It is reported that in 2020, the global incidence and mortality of gastric cancer are 11.1/100 000 and 7.7/100,000, respectively, making it the fifth most common malignant tumor in the world, second only to lung cancer, breast cancer and colon cancer in mortality rate, especially the highest incidence in East Asia [1]. At present, radical surgery remains the first choice for GC patients. According to a large survey in Japan in 2018, the 5-year overall survival (OS) rate of GC patients undergoing resection was 71.1% [2]. However, the early manifestation of GC is hidden, resulting in many patients being diagnosed as advanced stage and losing the opportunity of surgery, which not only has poor treatment effect, but also causes huge economic burden to patients and society [3, 4]. For these patients who have no chance of surgery at first, how to prolong survival through comprehensive treatment is a hot and difficult point in clinical research [5]. In recent years, the advent of conversion therapy has brought the possibility of surgical treatment for patients with advanced GC. Conversion therapy refers to the transformation of unresectable tumors into resectable ones through chemotherapy, radiotherapy, targeted therapy, immunotherapy, etc., prolonging the survival time and improving the quality of life. However, not all patients with advanced GC are candidates for conversion therapy. Those patients with the primary tumor and metastases cannot be resected by R0 (no residue under the microscope), including technically difficult to remove liver metastases, extensive group 16 lymph node metastasis, extensive peritoneal metastases, and tumors that invade adjacent organs and cannot be resected by R0, may opt for conversion therapy [6]. Growing evidence has suggested that the prognosis of patients with initial unresectable GC can be improved by conversion therapy. In 2017, a large retrospective analysis in Japan showed significant results in conversion therapy in patients with inoperable GC using a DCS chemotherapy regimen (docetaxel/cisplatin/S-1) with a median OS of up to 47.8 months [7].

In terms of conversion therapy, how to choose therapeutic drugs is equally crucial. At present, there is no unified standard for conversion therapy of GC. Many therapeutic regimes are selected based on the results of phase III clinical studies of advanced GC, such as a combination of platinum, fluorouracil and taxane.

**Fig. 1:**
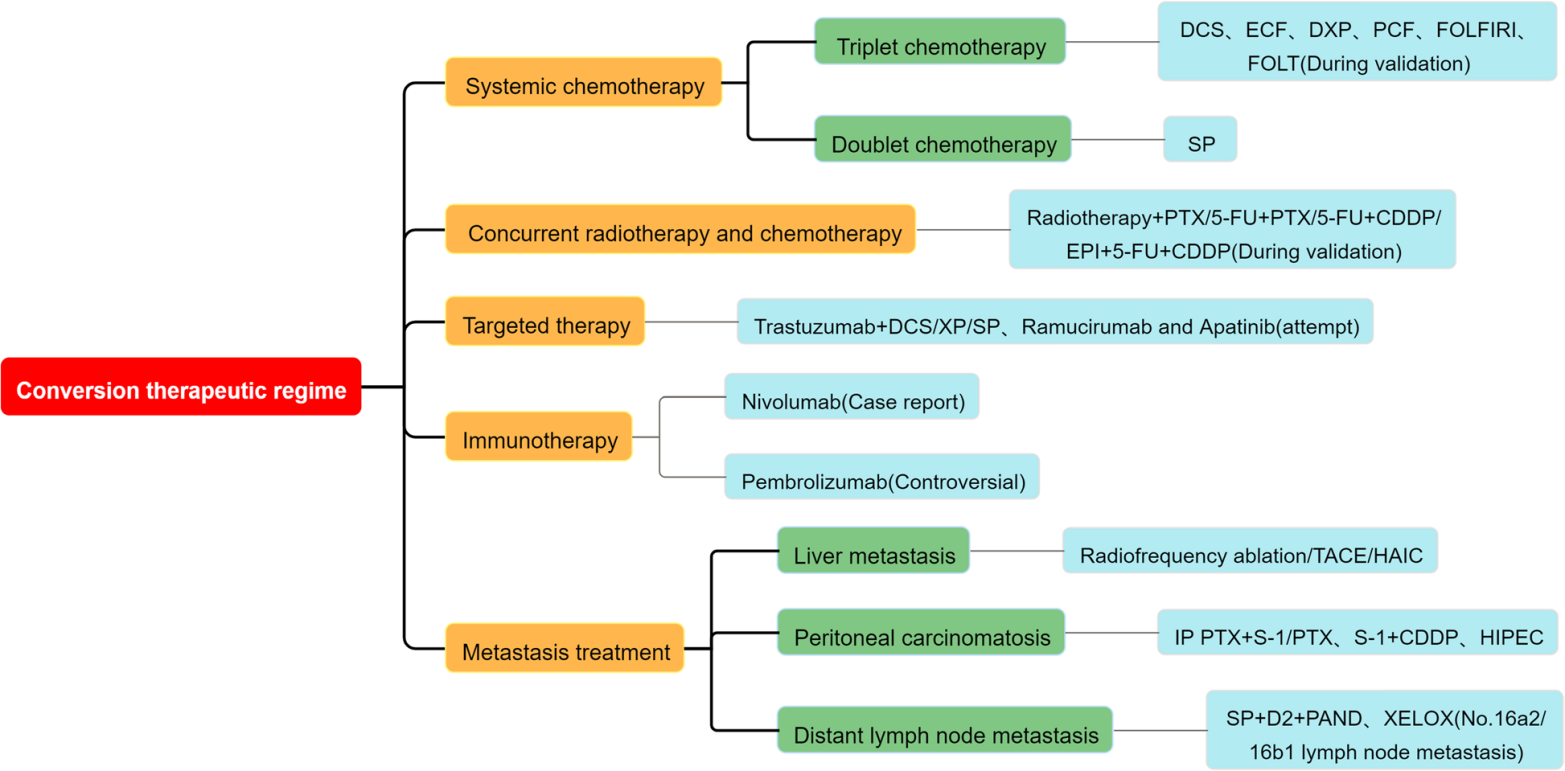
The regime of gastric cancer conversion therapy. DCS docetaxel, cisplatin, S-1; ECF epirubicin, cisplatin, 5-FU; DXP docetaxel, capecitabine, cisplatin; PCF PTX, Cisplatin, 5-FU; FOLFIRI 5-FU, leucovorin, irinotecan; FOLT 5-FU, oxaliplatin, leucovorin, docetaxel; SP S-1, cisplatin; CDDP cisplatin; EPI Epirubicin; IP irinotecan, cisplatin; TACE transcatheter arterial chemoembolization; HAIC Hepatic artery infusion chemotherapy; HIPEC hyperthermic intraperitoneal chemotherapy

In recent years, chemotherapy combined with radiotherapy, targeted therapy or immunotherapy, and local treatment for some metastatic lesions has gradually been discussed in the conversion therapy of advanced GC (Fig. 1). The research of Fukuchi et al indicated that compared with chemotherapy alone, conversion surgery after cisplatin combined with S-1 conversion therapy can significantly prolong the OS of patients (median OS, 53.0 versus 14.0 months, *p* < 0.01) [8]. However, little evidence supports the use of apatinib combined with chemotherapy in conversion therapy.

Apatinib is a small molecule tyrosine kinase inhibitor that selectively targets VEGFR-2, which can inhibit the formation of new blood vessels in tumor tissue, and then inhibit the growth of tumor [9]. Phase III clinical studies of apatinib have shown that apatinib can significantly prolong PFS and OS in patients with advanced or metastatic GC compared with placebo [10]. The research of Xu et al argued that apatinib improves the efficacy of 5-Fu both in vitro and in vivo [11]. Clinical evidence suggested that apatinib combined with chemotherapy may be an efficient and acceptable safety therapy for advanced GC, especially in conversion surgery [11]. In 2021 ASCO Annual Meeting, a research of Li et al about neoadjuvant/conversion therapy with camrelizumab, apatinib, S-1 ± oxaliplatin for locally advanced cT4a/bN+ gastric cancer showed that 79.2% of the 24 patients achieved the tumor descending stage. Of the 18 patients who underwent R0 resection, 44.4% achieved pathological remission [12]. Another study of Xia et al about apatinib plus DS chemotherapy regimen (docetaxel/S-1) in the first-line treatment of metastatic GC showed that the objective remission rates (ORR) of these patients was 60.47%, the disease control rates (DCR) was 81.4%, the median PFS was 7.46 months, the median OS was 12.42 months [13].

According to the above results, we selected unresectable patients with advanced GC and divided them into apatinib plus SOX regimen (S-1/Oxaliplatin) conversion therapy group and SOX regimen conversion therapy group. The baseline characteristics and survival data of the two groups were reviewed and analyzed. The purpose of this study was to evaluate the efficacy and safety of apatinib combined with SOX regimen in the conversion therapy of advanced GC.

## Patients and methods

### Patients

We reviewed the data of patients with unresectable gastric cancer treated in Zhejiang Provincial People's Hospital from June 2016 to December 2019, and conducted this retrospective cohort study. All patients received SOX regimen chemotherapy combined with or without apatinib-targeted treatment. The main inclusion criteria include (1) the patient was confirmed as gastric adenocarcinoma by pathological examination, and the clinical stage before treatment was evaluated by endoscopic examination and/or imaging evaluation (AJCC, 8th edition). (2) The patient must have one or more non-resectable factors, including invasion of adjacent tissues (T4), extensive lymph node metastasis (N3), liver metastasis (H1, the number of metastases < 3, total diameter of metastases < 5 cm), peritoneal metastasis (P1) or ascites cytology positive, and ovarian metastasis (O1). (3) The patient’s performance status of the Eastern Cooperative Oncology Group (ECOG-PS) is 0–2. (4) The patient has not experienced any anti-tumor treatment before diagnosis. The main exclusion criteria include patients with severe organ dysfunction, loss of follow-up and serious lack of information. This retrospective study was approved by the Local Ethics Committee of Zhejiang Provincial people's Hospital (Approval number: 2017KY012).

### Therapy schedule

Patients in SOX conversion group were treated with oral S-1 (40 mg/m^2^, twice a day) on days 1–14, and oxaliplatin (130 mg/m^2^) was given intravenously on day 1, every 21 days as a treatment cycle. Patients in SOX combined with apatinib conversion group continued to take apatinib (500 mg/day) on the basis of SOX regimen. When adverse reactions occurred, some patients adjusted dosages.

### Assessment

According to the Response Evaluation Criteria in Solid Tumors (RECIST) version 1.1, the tumor response was objectively evaluated by Computed tomography (CT) every 2–3 treatment cycles. The adverse events were evaluated according to the Common Terminology Criteria for Adverse Events (CTCAE) version 5.0. Meanwhile, the changes of CEA and CA19-9 at baseline, after conversion treatment and after surgery were evaluated by collecting data of relevant tumor markers in each treatment cycle. Conversion therapy is terminated when the disease progresses, serious adverse reactions or patients request to stop treatment. The deadline for data was June 25, 2021.

### Indications for conversion surgery

The important indication of conversion surgery is radical resection according to the therapeutic effect. For patients with disappearance of non-curative factors before conversion surgery, good response to chemotherapy, improvement or stability, the possibility of conversion surgery is evaluated through multi-disciplinary treatment (MDT), and if possible, radical resection is performed. If it cannot be resected or patients and their families refuse surgery or there are other reasons such as surgical contraindications, SOX chemotherapy alone will be continued.

### Statistical analyses

IBM SPSS Statistics version 26.0 (IBM Corp., Armonk, N.Y., USA) and GraphPad Prism version 5.0 (GraphPad Software, San Diego, CA, USA) were used for statistical processing. Patient characteristics were compared using the *χ*^2^ test or Fisher’s exact probability test. Through the normality test, the measurement data conform to the normal distribution, which is expressed by $\overline x\pm s$, and the non-normal distribution is expressed in median and range. *t* test was used for comparison between the two groups. Kaplan–Meier survival analysis was used to draw the survival curve, and compared survival curves by the log-rank test. The difference was statistically significant with *p* < 0.05.

## Results

### Clinical factors of patients

**Fig. 2:**
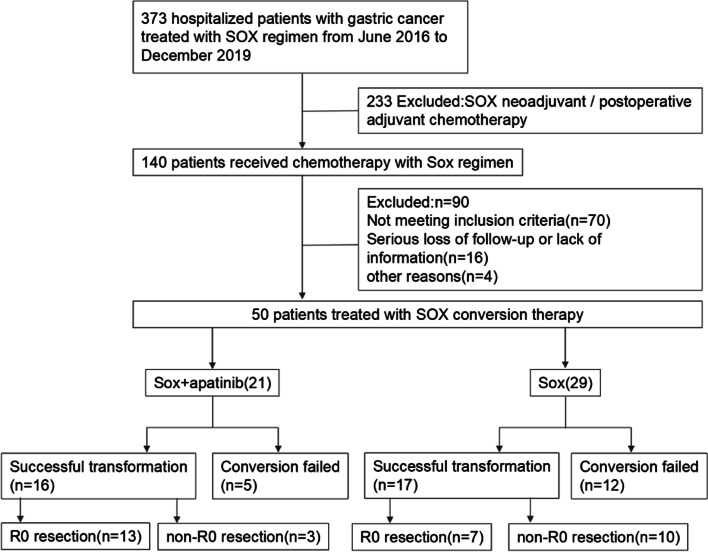
Flow diagram. SOX S-1/oxaliplatin

By the end of the study, a total of 50 patients were enrolled in the study. Figure 2 shows the process of patient enrollment. Table 1 shows the baseline characteristics of these 50 patients. Of these patients, there were 31 males and 19 females, with a median age of 64 years. 21 of these patients were in SOX combined with apatinib conversion group, other 29 patients in SOX conversion group. The whole conversion chemotherapy lasted about 3–6 cycles, and the median treatment time was 4 cycles. Apatinib was generally stopped 4 or 6 weeks before operation.

**Table 1:** Patient characteristics

Characteristic	Total, *n* (%)	The group of apatinib combined with SOX regimen in conversion treatment, *n* (%)	The group of SOX regimen in conversion treatment, *n* (%)	*P* value
Patients, *N* (%)	50	21	29	
Age (year) median (range)	64(50–76)	64(50–72)	64(51–76)	0.496
Sex				0.759
Male	31(62.0)	12(57.1)	19(65.5)	
Female	19(38.0)	9(42.9)	10(47.6)	
ECOG performance status				0.693
0	29(58.0)	11(52.4)	18(85.7)	
1	21(42.0)	10(47.6)	11(52.4)	
Location				0.603
Upper	15(30.0)	5(23.8)	10(47.6)	
Middle	20(40.0)	10(47.6)	10(47.6)	
Lower	15(30.0)	6(28.6)	9(31.0)	
Borrmann type				0.624
I	0(0.0)	0(0.0)	0(0.0)	
II	16(32.0)	7(33.3)	9(31.0)	
III	27(54.0)	10(47.6)	17(58.6)	
IV	7(14.0)	4(19.0)	3(10.3)	
Tumor depth				0.801
T2	0(0.0)	0(0.0)	0(0.0)	
T3	26(52.0)	12(57.1)	14(48.3)	
T4a	6(12.0)	2(9.5)	4(13.8)	
T4b	18(36.0)	7(33.3)	11(52.4)	
Nodal stage				0.979
N1	0(0.0)	0(0.0)	0(0.0)	
N2	13(26.0)	6(28.6)	7(24.1)	
N3	37(74.0)	15(71.4)	22(75.9)	
Peritoneal metastasis				0.849
No (P0)	46(92.0)	19(90.5)	27(93.1)	
Yes (P1)	4(8.0)	2(9.5)	2(6.9)	
Hepatic metastasis				0.913
No (H0)	42(84.0)	17(81.0)	25(86.2)	
Yes (H1)	8(16.0)	4(19.0)	4(13.8)	
Ovarian metastasis				0.613
No (O0)	50(100.0)	21(100.0)	29(100.0)	
Yes (O1)	0(0.0)	0	0(0.0)	
Clinical Stage				0.821
II	21(42.0)	8(38.1)	13(44.8)	
IVa	19(38.0)	8(38.1)	11(37.9)	
IVb	10(20.0)	5(23.8)	5(17.2)	
Peritoneal cytology				0.849
Negative	46(92.0)	19(90.5)	27(93.1)	
Positive	4(8.0)	2(9.5)	2(6.9)	
No. of non−curative factors				0.893
1	34(68.0)	14(66.7)	20(69.0)	
v2	16(32.0)	7(33.3)	9(31.0)	
Type of gastrectomy				0.902
Subtotal	11(22.0)	5(23.8)	6(20.7)	
Total	22(44.0)	11(52.4)	11(37.9)	
Postoperative chemotherapy				
Yes	33(66.0)	16(76.2)	17(58.6)	0.492
No	0(0.0)	0(0.0)	0(0.0)	

### Clinical effects analysis of apatinib

We first evaluated apatinib combined with SOX regimen conversion group and SOX regimen conversion group to research the role of apatinib in conversion surgery and the effect of targeted therapy combined with chemotherapy on the prognosis of inoperable GC patients. In the SOX plus apatinib group, 15 patients achieved partial remission (PR) and 4 patients received stable disease (SD). Thus, the ORR were 71.4% (15/21), and the DCR was 90.5% (19/21). In the SOX group, 16 patients achieved PR and 9 patient was SD, the ORR was 55.2% (16/29), the DCR was 86.2% (25/29). Although the ORR and DCR of SOX combined with apatinib treatment group were higher than those of SOX alone group, the difference was not statistically significant (Table 2). We statistically analyzed all patients who received conversion therapy, whether combination therapy or chemotherapy alone. The ORR and DCR of the conversion therapy were 62% (31/50) and 88.0% (44/50), respectively.

**Table 2:** preoperative chemotherapy response of patients who accepted two kinds of conversion chemotherapy regimen

Preoperative chemotherapy response	Apatinib plus SOX regimen, *n* (%)	SOX regimen, *n* (%)	*P* value
CR	0	0	0.497
PR	15 (71.4)	16 (55.2)	
SD	4 (14.3)	9 (31.0)	
PD	2 (9.5)	4 ((13.8)	
ORR	15(71.4)	16(55.2)	0.382
DCR	19(90.5)	25(86.2)	0.986

### Conversion rate analysis of apatinib

**Fig. 3:**
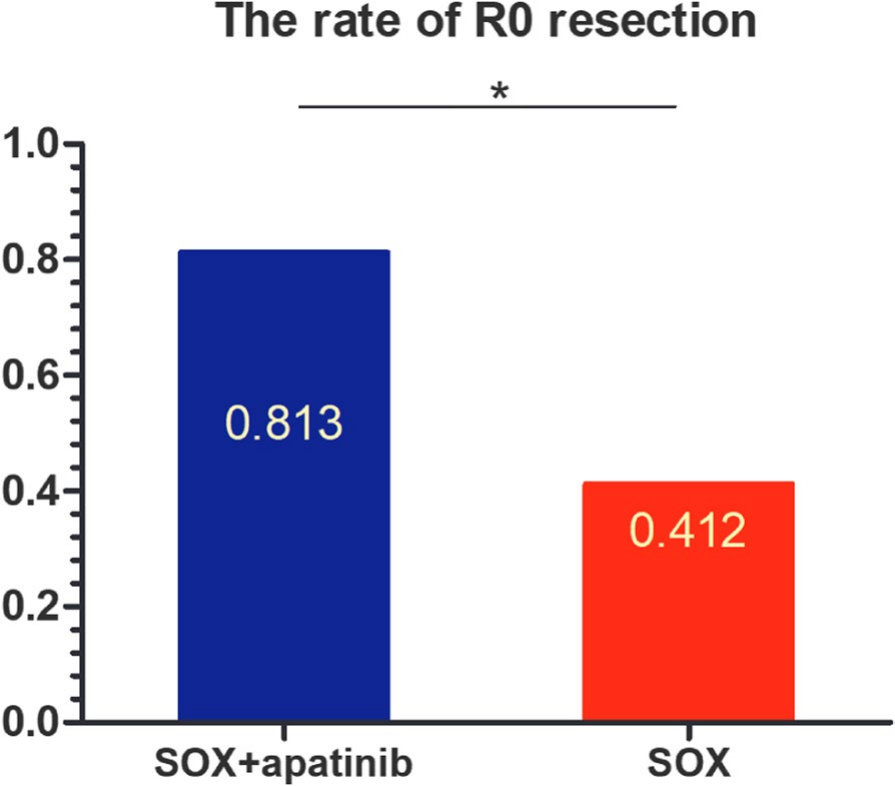
The R0 resection rates of patients who underwent conversion surgery (*p < 0.05)

Of all 50 patients, 33 underwent surgery, with an overall conversion rate of 66% (33/50). Among them, there were 16 patients in the SOX plus apatinib group, and the conversion rate was 76.2% (16/21), which was higher than 58.6% (17/29) in the SOX alone group. There was not significant difference between the two groups (*p* = 0.196). During the conversion operation, 13 patients in apatinib combined with SOX regimen conversion group achieved R0 resection of GC, 3 patients failed to achieve R0 resection, the R0 resection rates were 81.3% (13/16), while 17 patients in SOX regimen conversion group, R0 resection was achieved in 7 patients and 10 patients failed to achieve R0 resection, so the R0 resection rates were 41.2% (7/17). The R0 resection rate in SOX plus apatinib group was higher than that in SOX alone group, which was statistically significant (*p* = 0.032, Fig. 3).

**Fig. 4:**
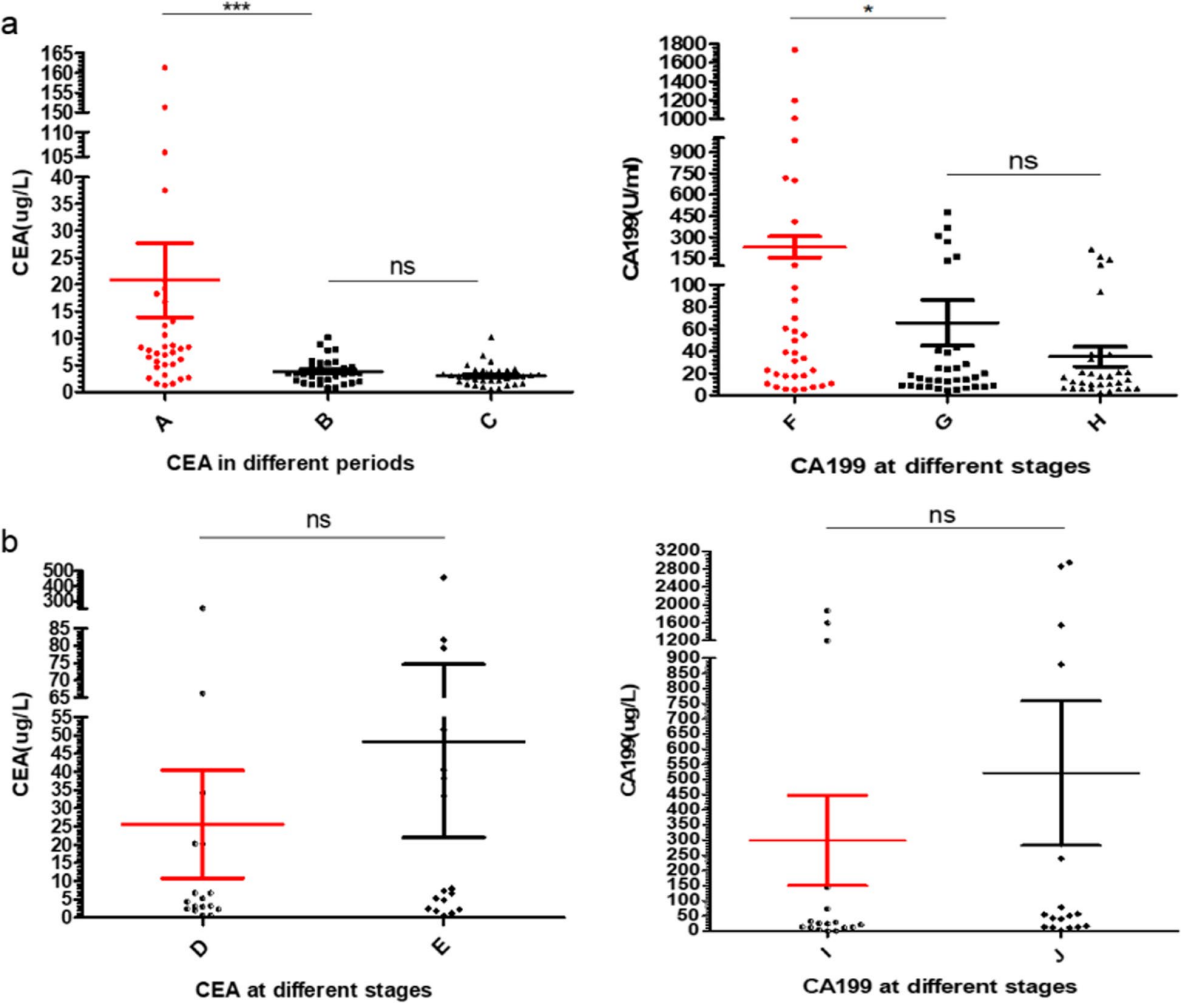
CEA and CA19-9 at different stages. **a** CEA and CA19-9 level change in the successful conversion group. A, B, and C represent the CEA level at baseline, before and after operation, respectively. F, G, and H represent the CA19-9 level at baseline, before and after operation, respectively. **b** CEA and CA19-9 level change in the failed group. D and E represent the CEA level at baseline and at the end of conversion therapy, respectively. I and J represent the CA19-9 level at baseline and at the end of conversion therapy, respectively. (***p < 0.001; ns, no significance)

In order to further analyze the curative efficacy of conversion therapy, we combined the patients who successfully underwent surgery after conversion therapy into the conversion therapy success group, and the patients who did not receive surgical treatment for various reasons were divided into the conversion therapy failure group. We analyzed the changes of tumor markers CEA and CA19-9 in conversion therapy success group and conversion therapy failure group respectively (Fig. 4). The results showed that after successful conversion therapy, compared with the baseline level, the level of CEA decreased significantly (*p* < 0.0001, Fig. 4a). Similarly, in conversion therapy success group, the CA199 level also decreased compared with the baseline level after conversion therapy (*p* = 0.045, Fig. 4a). However, in the conversion therapy failure group, compared with the baseline level, the tumor markers CEA and CA199 had an upward trend, but there was no statistical difference (*p* = 0.344; *p* = 0.242. Figure 4b).

### Survival analysis

**Fig. 5:**
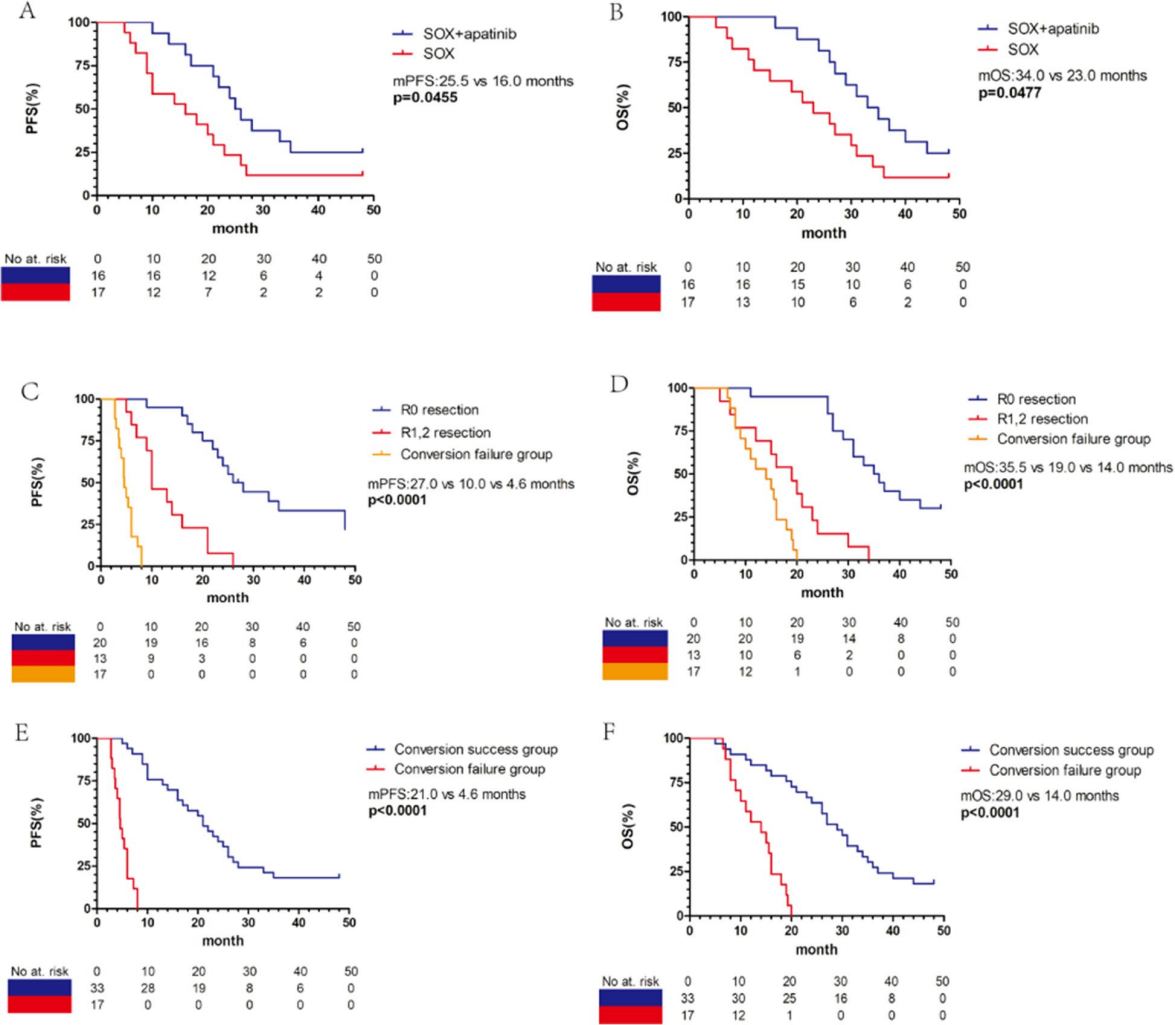
Kaplan–Meier survival curves. **A**, **B** PFS and OS between patients received SOX plus apatinib and SOX in conversion therapy. **C**, **D** PFS and OS of R0 resection, non-R0 resection and conversion failure group. **E**, **F** PFS and OS of conversion success group and conversion failure group. PFS, progression-free survival; OS, overall survival; SOX, oxaliplatin/S-1

Overall survival curves are shown in Fig. 5. In the conversion therapy success group, 16 patients received SOX combined with apatinib, while 17 patients received SOX alone. The median PFS of patients treated with SOX plus apatinib and SOX were 25.5 months (95% CI 21.1–28.9) and 16 months (95% CI 8.4–23.6), respectively (*p* = 0.0455, Fig. 5A); the median OS was 34.0 (95% CI 25.2–40.8) versus 23.0 (95% CI 16.3–29.7) months (*p* = 0.0477, Fig. 5B). Among the 33 patients treated with surgery, the median OS and PFS of 20 patients who underwent R0 resection were 35.5 months (95% CI 28.4–41.6) and 27 months (95% CI 19.8–32.2), respectively. The median OS and PFS of 13 patients who received non-R0 resection were 19 months (95% CI 14.8–23.2) and 10 months (95% CI 7.5–12.3), respectively. R0 resection resulted in significantly longer OS and PFS than non R0 resection (*p* < 0.0001 Fig. 5C, D). In order to evaluate whether conversion therapy can really bring survival benefits, we contrasted the survival data of the conversion therapy success group and conversion therapy failure group. The results showed that the median OS and PFS of patients in the conversion therapy success group were significantly longer than those in the conversion therapy failure group (median OS, 29.0, 95% CI 24.3–33.7 vs 14.0, 95% CI 10.2–17.8 months; *p* < 0.0001; median PFS, 21.0, 95% CI 15.4–26.6 vs 4.6, 95% CI 3.9–5.3 months; *p* < 0.0001, Fig. 5E, F).

### Safety

Adverse events (AEs) were assessed in 50 patients. All patients experienced different degrees of AEs. The common treatment-related side reaction in SOX combined with apatinib conversion group were vomiting (47.6%), neutropenia (42.9%), leucopenia (33.3%), and oral mucositis (23.8%), of which grade 3–4 adverse reactions accounted for 22.4% (11/49) of any grade adverse reactions. In the SOX conversion group, the common treatment-related AEs at any level were vomiting (48.3%), neutropenia (37.9%), leucopenia (31.0%), and 26.5% of patients suffered from 3–4 AEs. However, there was no significant difference between the two groups in the toxicity level of the conversion therapy regimen (Table 3). AEs associated with apatinib treatment include oral mucositis (23.8%), hypertension (19.0%) and hand-foot syndrome (19.0%), of which the incidence of serious AEs is low, including hypertension (*n* = 1, 4.8%) and hand-foot syndrome (*n* = 1, 4.8%).

**Table 3:** Incidence of treatment-related adverse events of two treatment regimens during preoperative treatment

Adverse events	SOX + apatinib (*N* = 21)		SOX (*N* = 29)		*P* value
	Any grade, *n* (%)	Grade 3/4, *n* (%)	Any grade, *n* (%)	Grade 3/4, *n* (%)	
Neutropenia	9 (42.9%)	3 (14.3%)	11 (37.9%)	4 (13.8%)	0.731
Leukopenia	7 (33.3%)	2 (9.5%)	9 (31.0%)	3 (10.3%)	0.712
Thrombocytopenia	3 (14.3%)	1 (4.8%)	4 (13.8%)	2 (6.9%)	0.673
Hypertension	4 (19.0%)	1 (4.8%)	0	0	0.777
Hand-foot syndrome	4 (19.0%)	1 (4.8%)	0	0	0.777
Oral mucositis	5 (23.8%)	0	0	0	0.915
Vomiting	10 (47.6%)	2 (9.5%)	14 (48.3%)	3 (10.3%)	0.667
Peripheral neuropathy	3 (14.3%)	0	5 (17.2%)	0	0.600
Fatigue	4 (19.0%)	1 (4.8%)	6 (20.7%)	1 (3.4%)	0.600

## Discussion

In the past, systemic chemotherapy was considered to be one of the best choices for patients with advanced GC who had no chance of operation for the first time [14]. But new researches indicate that conversion surgery may lead to further long-term survival, and patients who receive conversion therapy survive longer than those who receive chemotherapy alone [5–8, 15]. ESMO Clinical Practice Guidelines also advices that gastrectomy should be actively considered in order to improve the prognosis of patients with good response to systemic chemotherapy [16]. In the research of Professor Fukuchi M et al, the median OS of patients receiving conversion therapy was 37 months, while the median OS of patients who only received chemotherapy was less than 1 year [17]. Kim SW et al. studied GC patients with peritoneal metastasis and discovered that the survival time of those sufferers with complete resection was greatly improved after successful conversion therapy [18]. In this study, more patients in the successful conversion treatment group received PR during preoperative chemotherapy, and the tumor markers CEA and CA199 were significantly reduced. Meanwhile, the median OS and PFS in the successful conversion therapy group were significantly longer than those in the failed conversion therapy group, indicating that patients with advanced inoperable GC could indeed achieve long-term survival from successful conversion therapy.

At present, SOX regimen is mainly used for neoadjuvant therapy and postoperative adjuvant chemotherapy for GC [19]. The use of SOX regimen in the conversion therapy of initial unresectable GC is rare in the literature. Apatinib is only used for the third-line treatment of GC in most cases. Related studies have shown that the combination of anti-angiogenic-targeted drugs and chemotherapy can produce synergistic effect and improve the efficacy of chemotherapy [11, 20, 21]. Our previous case reports have revealed the effectiveness of apatinib combined with SOX regimen chemotherapy in the conversion treatment of advanced GC [22]. However, at present, there is no research report comparing the efficacy and safety of SOX combined with apatinib and SOX alone in the conversion therapy of GC. Our retrospective study shows that the ORR and DCR of the SOX conversion group are 55.2% and 86.2% respectively. The ORR and DCR of the SOX combined with apatinib conversion group are 71.4% and 90.5% respectively. Obviously, apatinib enhances the anti-tumor response of SOX regimen in conversion therapy, and improves the efficacy.

Surgery is the foundation stones of radical cure of GC, and R0 resection is the goal of surgical treatment. Compared with non-R0 resection, R0 resection can significantly improve the survival time of patients. In our study, the conversion rate of SOX combined with apatinib was 76.2%, and the R0 resection rate was 81.3%. The work of Xu et al. included 31 patients with advanced GC who received apatinib combined with PTX/S-1 conversion therapy, with a conversion rate of 60% and a R0 resection rate of 56.7% [23]. Obviously, our results are better. This may be related to the following reasons: Firstly, the proportion of peritoneal metastasis in our patients (9.5%) is less than that in Xu et al.’s study (29.0%). Peritoneal metastasis is considered to be related to the lower conversion rate and R0 resection rate of patients [24]. Secondly, no patients with ovarian metastasis were included in this study. The prognosis of ovarian metastasis from gastric cancer is worse than that of ovarian metastasis from other gastrointestinal sources. The median survival time is only 7–14 months [25], which is one of the main reasons for treatment failure of female gastric cancer patients. However, it is difficult to distinguish primary ovarian tumor and metastatic tumor, especially when the tumor is small and the symptoms are not obvious. In addition, some benign ovarian tumors may also have abdominal tumors, ascites, pleural effusion and the increase of tumor marker CA125, which strongly suggests the symptoms of disseminated malignant tumors [26]. Therefore, depending solely on the clinical manifestation of patients and the increase of tumor biomarkers does not seem to be able to distinguish ovarian masses. Calster et al. integrated clinical predictors (age, serum CA-125 level, type of center) and ultrasound predictors to distinguish primary and metastatic ovarian tumors [27]. Zaccaria et al. even confirmed this diagnosis by laparoscopic surgical resection and pathological examination [28]. In the future, it may further improve the success rate of conversion therapy of gastric cancer patients by combining multiple methods to diagnose primary ovarian tumor or metastatic tumor and stratify patients suitable for transformation treatment. Finally, many of our patients (68.0%) have only one unresectable factor. In the multicenter retrospective study conducted by Sato et al., most of the 33 patients with advanced GC who successfully underwent conversion surgery after DCS conversion therapy had only one unresectable factor, and the R0 resection rate reached 84.8% [7]. This is similar to our research results.

In terms of adverse reactions, Lin et al. indicated that the most common hematological adverse reactions of apatinib combined with SOX regimen were neutropenia and leukopenia, and the common adverse reactions associated with apatinib treatment were hypertension, hand-foot syndrome and albuminuria. The combination of the two did not increase the incidence of grade 3 or more adverse reactions and preoperative mortality in patients [11]. Another small sample study also shows that apatinib can be safely used in combination with SOX regimens [29]. In this research, SOX regimen generally used 3–6 cycles and a median of 4 cycles. The most common adverse reactions observed were vomiting, neutropenia and leukopenia, but most of them occurred in grade 1–2. These events were effectively controlled after symptomatic treatment, and no serious adverse events, especially gastrointestinal perforation and bleeding, were observed. The overall safety results are roughly the same as those of the above two studies. In summary, our data show that preoperative apatinib-targeted therapy combined with chemotherapy is considered safe under proper management.

Conversion therapy is a new treatment concept, the literature reference of conversion therapy is still insufficient. We have provided an idea for the choice of conversion treatment, but this study is a retrospective study, the number of patients involved is relatively small, and there is a certain selection bias in the process of sample selection. A large sample, multi-center randomized controlled trials are still needed to verify our results.

## Conclusion

Patients with advanced inoperable gastric cancer could benefit from conversion chemotherapy and subsequent conversion surgery, and apatinib-targeted therapy combined with SOX chemotherapy may be a safe and feasible option for conversion therapy.

## Data Availability

The original contributions presented in the study are included in the article/supplementary material; further inquiries can be directed to the corresponding authors.

## Abbreviations

GC: Gastric cancer
PFS: Progression-free survival
OS: Overall survival
MDT: Multi-disciplinary treatment
ASCO: American Society of Clinical Oncology
AJCC: American Joint Committee on Cancer
ESMO: European Society for Medical Oncology
